# A Rare Adolescent Presentation of Morvan Syndrome: Diagnostic Challenges and Therapeutic Response

**DOI:** 10.1002/ccr3.72122

**Published:** 2026-02-19

**Authors:** Muhammad Umar, Majeed Ullah, Umm E. Salma Shabbar Banatwala, Ayesha Afridi, Umama Alam, Okasha Tahir, Fatima Sajjad, Muhammad Abdullah Ali, F. N. U. Noormal, Jibran Ikram

**Affiliations:** ^1^ Khairpur Medical College Khairpur Pakistan; ^2^ Hayatabad Medical Complex Peshawar Pakistan; ^3^ Dow University of Health Sciences Karachi Pakistan; ^4^ Riphah International University Islamabad Pakistan; ^5^ Khyber Medical College Peshawar Pakistan; ^6^ Bacha Khan Medical College Mardan Pakistan; ^7^ Cleveland Clinic Foundation Cleveland Ohio USA

**Keywords:** autoimmune encephalopathy, autoimmune neurological disorder, immunotherapy response, Morvan syndrome

## Abstract

Morvan syndrome is a rare autoimmune disorder that predominantly affects adults, but it can also present in adolescents with atypical symptoms. This case of a 17‐year‐old patient exhibited severe back pain, muscle twitching, visual hallucinations, and hyponatremia, without the thymoma or malignancy commonly observed in adult cases. Early diagnosis and prompt immunotherapy with corticosteroids and plasmapheresis resulted in significant symptom improvement, highlighting the importance of timely intervention in complex neuroimmunological disorders.

## 1 Background

Morvan syndrome is a rare autoimmune neurological disorder, characterized by neuromyotonia, dysautonomia, encephalopathy, and severe insomnia. It is a life‐threatening condition, affecting fewer than 1 in a million individuals globally [1, 2]. This syndrome is associated with antibodies targeting proteins in the voltage‐gated potassium channel (VGKC) complex.

Although numerous case studies describe patients with Morvan syndrome, the average age of onset is typically between 37 and 66 years [3, 4, 5]. Pediatric and adolescent cases remain rare and underrepresented. Here, we report an atypical early‐onset case in a 17‐year‐old male, highlighting age‐related variability in clinical presentation, antibody profile, and treatment response. This case offers valuable insights in managing such presentations.

## 2 Case Presentation

A 17‐year‐old male presented with a one‐month history of severe back pain, unresponsive to standard analgesics, progressing to lower limb pain, fatigue, and episodic falls. Within 3 weeks, he developed generalized body aches, high‐grade fever (103°F), and persistent muscle twitching affecting multiple muscle groups, including facial muscles. He also experienced visual hallucinations but remained cognitively intact.

**FIGURE 1:**
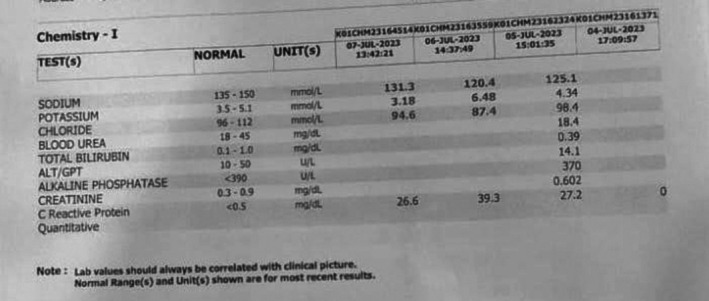
Serial laboratory results showing hyponatraemia.

Comprehensive investigations were performed. Laboratory findings revealed:

- Elevated total leukocyte count, particularly neutrophils.
- Mildly elevated hematocrit (35.2) and mean corpuscular hemoglobin concentration (36.9)
- Markedly low serum sodium as shown in Figure 1.

Peripheral smear and bone marrow biopsy were normal, and spinal MRI showed no abnormalities. Further tests, including chest X‐ray, abdominal ultrasound, and blood and urine cultures, were unremarkable.

## 3 Differential Diagnosis, Investigations, and Treatment

**FIGURE 2:**
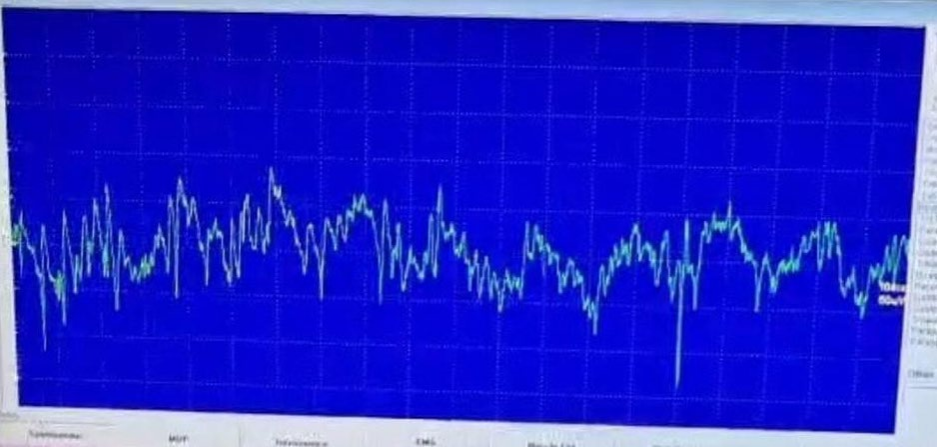
Electromyography (EMG) tracing showing myokymic discharges.

Electromyography revealed myokymic discharges (Figure 2).

**FIGURE 3:**
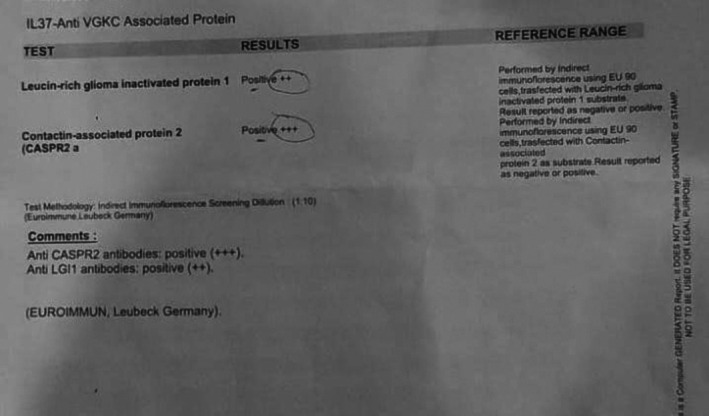
Serum autoimmune antibody testing showing positive serum CASPR2 and LGI1 antibodies.

Serum autoimmune antibody testing was performed using indirect immunofluorescence with EU90 cells. This demonstrated positivity for antibodies against the VGKC complex, with strong anti‐contactin‐associated protein‐2 (CASPR2) antibody positivity (+++) and moderate anti‐leucine‐rich glioma‐inactivated protein‐1 (LGI1) antibody positivity (++) (Figure 3).

A computed tomography (CT) scan of the thorax, abdomen, and pelvis (CT‐TAP) was performed to rule out thymoma or malignancies, both of which were absent. A positron emission tomography (PET) scan was not performed due to financial constraints. As CT‐TAP is typically used as the primary imaging modality for malignancy screening in resource‐limited settings.

Hyponatremia was evaluated in the context of euvolemia and normal renal, adrenal, and thyroid function, supporting a diagnosis of syndrome of inappropriate antidiuretic hormone secretion (SIADH). This finding is well described in association with LGI1 antibodies and likely contributed to the patient's electrolyte disturbance [6].

Differential diagnoses considered:

- Isaac syndrome: Overlapping features of peripheral nerve hyperexcitability but lacking central/autonomic dysfunction [7].
- Limbic encephalitis & isolated neuromyotonia: Limbic encephalitis more commonly presents with memory impairment, seizures, and confusion, whereas Morvan syndrome is associated with preserved cognition and more frequent hallucinations [6, 8] Although the patient demonstrated neuromyotonic features on electromyography, the presence of central nervous system manifestations, autonomic dysfunction, and LGI1 antibody positivity argues against isolated neuromyotonia and supports a diagnosis of Morvan syndrome [9].

Taken together, the coexistence of peripheral nerve hyperexcitability on EMG, central nervous system manifestations, autonomic dysfunction, and preserved cognition favors Morvan syndrome.

## 4 Treatment and Outcome

- Intravenous methylprednisolone (1 g/day for 5 days) and plasmapheresis (5 sessions on alternate days) led to significant improvement. Muscle stiffness and pain resolved initially, while remaining symptoms resolved subsequently.
- Discharged with:
  ○ Pregabalin (100 mg/day).
  ○ Oral prednisone (50 mg/day, tapered over 8 weeks).
  ○ Azathioprine (50 mg/day initially, increased to 100 mg/day).
  ○ Supportive therapy (proton pump inhibitors, calcium, and vitamin D).

## 5 Results

Three months post‐treatment, the patient remained stable, with fully tapered steroids and no recurrence of symptoms. His case highlights that Morvan syndrome can manifest atypically in adolescents, and early intervention with immunotherapy can lead to favorable outcomes.

## 6 Discussion

This case showcases an uncommon presentation of Morvan syndrome, emphasizing the critical importance of prompt recognition and assertive management. Unlike most reported cases occurring in middle‐aged adults, this case illustrates an early‐onset presentation with atypical pain and neuropsychiatric features. Presenting during adolescence, with visual hallucinations and intense, persistent back pain that initially resembled other conditions, this case highlights the variability in Morvan syndrome's symptomatology. The patient's rapid improvement following immunotherapy further supports the efficacy of early intervention in managing complex neuroimmunological disorders. Notably, this patient did not exhibit weight loss or severe insomnia, which are frequently reported in Morvan syndrome [10]. Plasmapheresis was selected as the treatment modality in this case due to its availability within our healthcare setting. Intravenous immunoglobulin was not administered because of financial constraints.

Notably, this patient exhibited no thymoma, which is often seen in Morvan syndrome and distinguishes it from classical limbic encephalitis [2, 11]. Previous reports indicate that Morvan syndrome can also be linked to other tumors, including lung cancer, testicular cancer, and lymphoma [12]. The coexistence of CASPR2 and LGI1 antibodies likely explains the overlap of peripheral nerve hyperexcitability, central nervous system manifestations, and SIADH, while strengthening the diagnostic rationale [5, 6, 8].

In formulating this diagnosis, we considered Isaac syndrome as a primary differential due to its overlapping feature of peripheral nerve hyperexcitability. However, central or autonomic nervous dysfunction, the key to Morvan syndrome, aids in distinguishing between the two [7]. Other potential differentials included limbic encephalitis and neuromyotonia, though Morvan syndrome typically presents with fewer instances of amnesia, confusion, seizures, and more frequent hallucinations compared to these conditions [8].

## 7 Conclusion

This case highlights an unusual presentation of Morvan syndrome in adolescence, demonstrating that the syndrome can manifest with atypical symptoms, including severe back pain and visual hallucinations without cognitive impairment. Early diagnosis and prompt initiation of immunotherapy, such as corticosteroids and plasmapheresis, can lead to substantial symptom improvement and overall stabilization, underscoring the importance of rapid, aggressive intervention in managing neuroimmunological disorders.

The absence of thymoma in this patient also emphasizes that Morvan syndrome can present without classical features, necessitating a thorough evaluation to distinguish it from other disorders, like Isaac syndrome, which share similar peripheral nerve hyperexcitability but lack central or autonomic dysfunction. This case contributes to the growing body of evidence on Morvan syndrome and highlights the need for awareness of age‐related variations in its clinical presentation, which may inform timely and effective treatment strategies for future cases.

## Author Contributions

**Muhammad Umar:** writing – original draft. **Majeed Ullah:** data curation. **Umm E. Salma Shabbar Banatwala:** supervision. **Ayesha Afridi:** writing – review and editing. **Umama Alam:** conceptualization. **Okasha Tahir:** visualization. **Fatima Sajjad:** methodology. **Muhammad Abdullah Ali:** resources. **F. N. U. Noormal:** writing – review and editing. **Jibran Ikram:** writing – review and editing.

## Funding

The authors have nothing to report.

## Consent

“Written informed consent” was obtained from the patient(s) to “publish” this report in accordance with the journal's patient consent policy. In the form, the patient has given his consent for his images and other clinical information to be reported in the journal. The patient understands that his name and initials will not be published, and due efforts will be made to conceal his identity.

## Conflicts of Interest

The authors declare no conflicts of interest.

## Data Availability

The data that support the findings of this study are available on request from the corresponding author. The data are not publicly available due to privacy or ethical restrictions.
